# A Case of Delayed Right Ventricular Pacemaker Lead-Associated Perforation Causing Cardiac Tamponade

**DOI:** 10.7759/cureus.91624

**Published:** 2025-09-04

**Authors:** Yuhi Nakamura, Motohiro Kawauchi, Yuki Tanaka

**Affiliations:** 1 Department of Cardiovascular Surgery, Kanto Rosai Hospital, Kawasaki, JPN

**Keywords:** cardiac perforation, cardiac tamponade, delayed pacemaker lead perforation, permanent pacemaker complication, right ventricular perforation

## Abstract

Right ventricular perforation by pacemaker leads is often reported. However, reports of delayed lead perforation, defined as the perforation that occurs >1 month after implantation, are rare. Despite the fact that delayed pacemaker lead perforation can be an emergency, no clear treatment strategy has been defined.

Herein, we report our experience with a case of delayed lead-associated perforation causing cardiac tamponade. A 60-year-old man presented to the emergency room with chest and back pain and dyspnea. He had undergone pacemaker implantation for advanced atrioventricular block eight years ago. He was found to have cardiac tamponade due to right ventricular lead-associated perforation. Emergency surgery was performed to drain the fluid and control the bleeding via a median sternotomy. The lead screw had perforated a vein on the cardiac surface. The perforating lead was retained because the threshold and impedance of the lead were unchanged. Only the perforation was repaired. After seven days, the ventricular lead threshold increased. This was managed by adjusting the pacemaker settings. The patient tolerated the procedure well and was discharged home. The treatment of right ventricular lead-associated perforation generally entails removal and reinsertion of the lead. However, in our patient, a definitive diagnosis could not be made before the operation because the right ventricle was not completely perforated by the lead tip. Furthermore, the patient had concomitant cardiac tamponade with signs of shock. Therefore, open surgery was performed. After the perforation site is repaired, the area around the myocardium may deteriorate for several reasons (e.g., edema, fibrosis, and partial myocardial tissue necrosis). Therefore, if the pacemaker lead cannot be removed (or reinserted), as in our patient, measures such as insertion or implantation of a temporary pacemaker lead need to be taken in anticipation of deterioration of the myocardium around the retained lead.

## Introduction

Hundreds of thousands of pacemaker implantations are performed worldwide each year [1]. Right ventricular lead-associated perforation develops in 0.1%-0.8% of patients following pacemaker implantation and in 0.6%-5.2% of patients with an implantable cardioverter-defibrillator (ICD) [2]. Acute perforation is defined as a perforation that occurs within the first 24 hours after implantation. Subacute perforation is defined as that occurring within 1-30 days, and delayed perforation is deﬁned as that occurring >30 days after implantation. Although right ventricular lead-associated perforation is often reported, reports of delayed lead-associated perforation are rare. According to several reports, the incidence of delayed lead-associated perforation has been reported as 0.04%, and the incidence of lead-related perforation occurring more than one year after implantation has been reported as less than 0.02% [3,4]. Potential risk factors for lead perforation include hard leads, thin tips, steroid medication, and low body weight. In our patient, a screw-in lead was used, and the lead was strongly bent. Accumulation of stress between the lead tip and the myocardium due to heartbeat was considered to have caused gradual penetration toward the myocardium, eventually leading to perforation. Scattered reports have stated that the amount of bleeding from the heart is not large, and patients are often hemodynamically stable [5-10]. However, delayed lead-associated perforation may result in an emergency. Treatment strategies for right ventricular lead-associated perforation are not clearly defined, and they differ from case to case. Depending on the clinical situation, management options such as open surgery (median sternotomy or lateral thoracotomy via an intercostal approach) with pacing lead replacement, or pericardiocentesis with lead replacement, have been employed, with the decision influenced by multiple factors, including the interval from pacemaker implantation to perforation and the patient’s vital signs [5-10]. This case report is one of the few reporting tamponade many years after implantation. Thus, we have reported our experience with a case of delayed lead-associated perforation that caused cardiac tamponade and required emergency surgery after eight years of implantation.

## Case presentation

**Figure 1:**
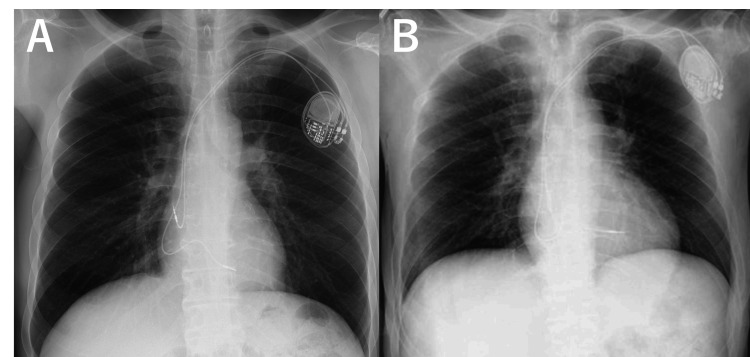
Chest radiographs (A) Taken one year before the current presentation. (B) Taken at the time of the current presentation, showing pulmonary congestion, which may reflect cardiac tamponade. In both radiographs, the pacemaker lead appears markedly bent.

**Figure 2:**
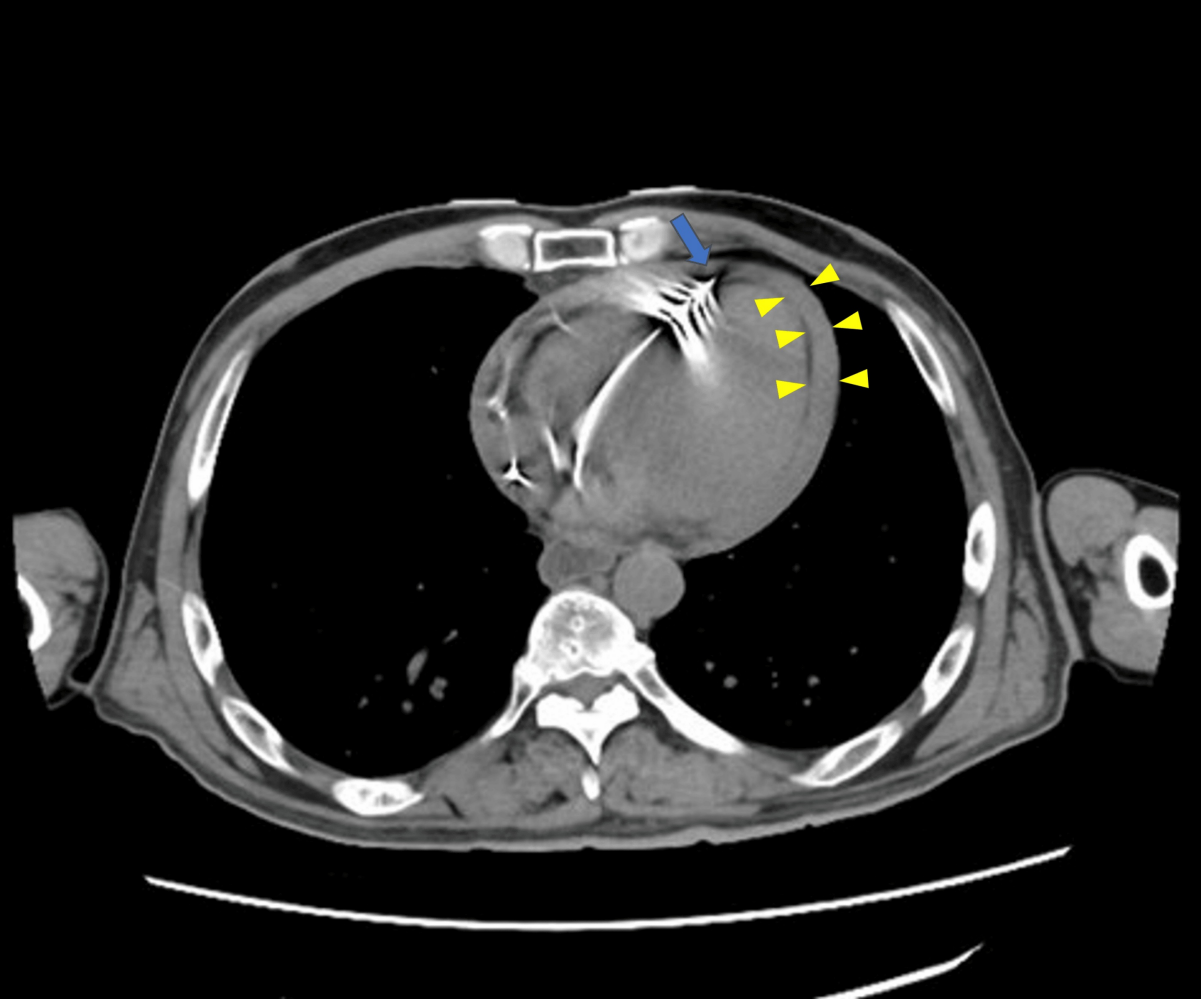
CT demonstrated deep penetration of the right ventricular lead into the right ventricular wall, although the presence of complete perforation was unclear (blue arrow); circumferential pericardial effusion around the heart was also observed in the pericardial space (yellow triangles) CT: computed tomography, RV: right ventricle

A 60-year-old man presented to the emergency room with symptoms of chest and back pain and dyspnea at rest. Chest tightness suddenly occurred at 1:00 a.m. on a certain day, and his sleep was intermittent that night. The chest tightness progressed to chest pain after hours, and the symptoms worsened. He called for emergency medical service nine hours after the first symptom occurred. He had undergone pacemaker implantation for advanced atrioventricular block eight years ago with screw-type atrial (CapSureFix Novus 5076-52; Medtronic, Minneapolis, MN) and ventricular (CapSureFix Novus 5076-58; Medtronic, Minneapolis, MN) leads. His medical history included hypertension, dyslipidemia, diabetes, and angina pectoris. He did not have a history of chest trauma. He was conscious, and his blood pressure was 65/45 mmHg on arrival, requiring high-volume vasopressor support. The electrocardiogram (ECG) revealed a heart rate of 87 bpm and no pacing failure. The lead’s threshold and impedance were similar to a recent examination’s findings (atrial lead: sense 1.5mV, threshold 0.75 V, and impedance 342 Ω; ventricular lead: sensing not detected, threshold 0.75 V, and impedance 418 Ω). As shown in Figure 1, chest radiography shows congestion, and the pacemaker lead was strongly bent. Echocardiography revealed a pericardial effusion. Figure 2 shows that computed tomography (CT) revealed circumferential accumulation of fluid in the pericardium and deeper than normal penetration of the right ventricular pacemaker lead into the myocardium of the anterior wall, although it was unclear whether the lead tip had traversed the epicardium. The pacemaker was functioning normally, and the patient’s cardiac rhythm was entirely pacemaker-dependent. As the patient was hemodynamically unstable and in a state of acute kidney injury, contrast-enhanced CT could not be performed.

**Figure 3:**
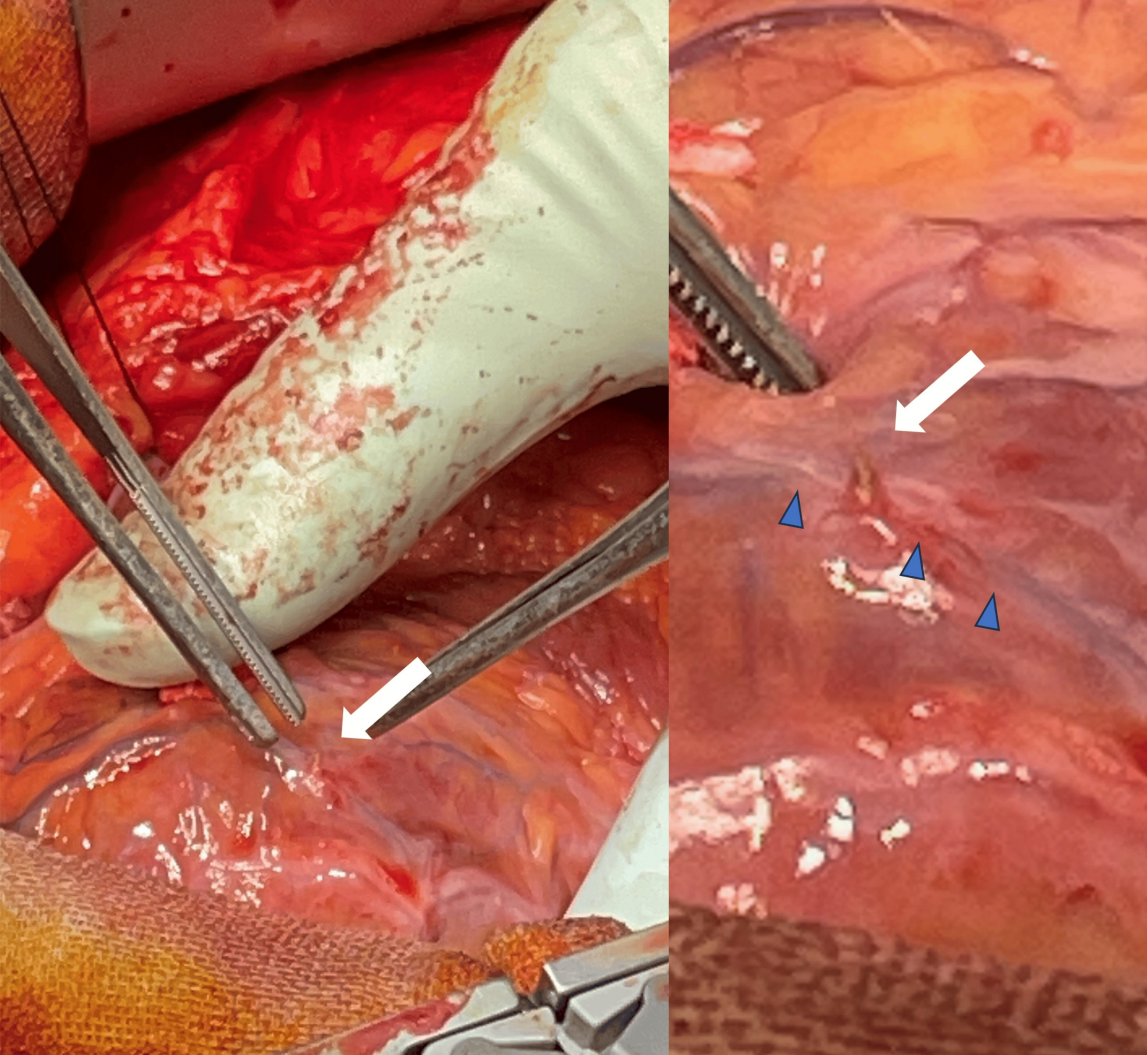
Intraoperative findings The RV lead tip protruded through the RV wall (white arrow), and it penetrated a vein on the cardiac surface (blue triangle). RV: right ventricle

Blood tests also revealed highly elevated hepatobiliary enzymes, which indicated a shock liver. The hemorrhagic pericardial fluid was drained by pericardiocentesis, but it was difficult to drain more than approximately 100 mL, and improvement in vital signs was limited. Thus, emergency open surgery and pericardiotomy were performed to release the cardiac tamponade and stop the bleeding, three hours after arrival, and 300 mL of hemorrhagic pericardial fluid was drained, consisting predominantly of liquid with only a small amount of clot. We confirmed the protuberance of the screw-in lead tip approximately 2-3 mm high from the anterior surface of the right ventricle. Figure 3 shows that there was no bleeding from the cardiac cavity, and the lead had penetrated a vein on the cardiac surface. This finding was consistent with the presence of cardiac tamponade despite the absence of clear findings indicating myocardial perforation on preoperative CT. The innominate vein was severely sclerosed and adherent; therefore, we could not remove the lead to avoid injury to the vein. However, the perforation site was sutured with a 2-0 braided polyester suture with felt pledgets.

**Figure 4:**
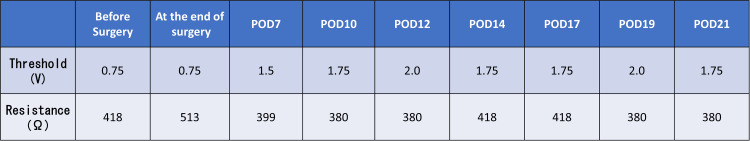
Ventricular lead threshold increased until POD 12, after which it stabilized POD: postoperative day

The pacemaker parameters at the end of surgery were unchanged from the preoperative values (atrial lead: sense 1.5mV, threshold 0.75 V, and impedance 361 Ω; ventricular lead: sensing not detected, threshold 0.75 V, and impedance 513 Ω), and the device was functioning normally, as it had before surgery. Therefore, placement of a temporary epicardial pacing lead was not performed. Postoperatively, the patient’s cardiac rhythm remained entirely pacemaker-dependent. However, seven days after surgery, the ventricular lead threshold had increased, resulting in transient pacing failure, which could be managed by adjusting the pacemaker settings. On the same day, a CT was performed to follow up on the position of the pacing lead, confirming no significant change from the preoperative status. In addition, echocardiography confirmed that myocardial wall motion was preserved; however, the occurrence of pacing failure was unforeseen. As shown in Figure 4, the RV lead threshold continued to rise until postoperative day 12, after which it stabilized. This increase in threshold was managed by adjusting the pacemaker settings. The patient tolerated the procedure well and was discharged home 24 days after the surgery. This patient is attending our outpatient visits. An additional RV lead was implanted, and it is working with no problems.

## Discussion

Right ventricular perforation develops in 0.1%-0.8% of patients with an implanted pacemaker and 0.6%-5.2% of patients with an ICD. The symptoms of lead-associated perforation include chest pain, abdominal pain, dyspnea, dizziness, malaise, and syncope. The risk factors of lead-associated perforation include stiff leads, thin tips, lead placement in the apex of the heart, age, female gender, steroid medication, and low body weight [11]. Hirschl et al. reported that asymptomatic right ventricular lead-associated perforation can be detected in 15% of CTs performed in patients with pacemakers or ICDs [12]. Sometimes, they are incidentally found during the examination of other diseases or treatments [13,14]. Occasionally, asymptomatic lead-associated perforations may not exhibit any abnormal electrophysiological parameters. Rajkumar et al. reported that in a blinded expert review of imaging studies, the sensitivity and specificity of CT were 100% and 85.7%, respectively, which demonstrates its excellent ability to diagnose cardiac perforations. The sensitivity and specificity of transthoracic echocardiography were 41.2% and 84.2%, respectively, while those of chest X-ray were 27.7% and 94.4%, respectively. Thus, they recommended obtaining an ECG-gated non-contrast CT scan with 2.5-mm slices of the heart and thoracic cavity if lead perforation is suspected [6].

Delayed perforation is defined as a perforation that develops >1 month after implantation. However, the development of pacemaker lead-associated perforation several years after implantation is rare. Issa et al. reported 54 cases of subacute (one day to one month after implantation) or delayed lead-associated perforation from their institution over a 13-year period, with time-to-perforation ranging from two to 412 days [11]. Cano et al. also reported 17 cases of right ventricular perforation in a prospective observation of 2,200 patients over a seven-year period. The time-to-subacute (or later) perforation ranged from seven to 15 days [15]. Lin et al. followed 36,104 patients for three years and reported an incidence of delayed pacemaker lead perforation of 0.04% [3]. Waddingham et al. reported that, among 10,631 cardiovascular implantable electronic device implantations, only two cases were diagnosed as lead perforation more than one year after implantation [4]. Among the eight reported cases of long-delayed right ventricular lead-associated perforation (>1 year after implantation), four presented with pericardial effusion, three underwent open chest surgery, and only one presented with cardiac tamponade [5-10] (Table 1). Our patient was the second case presenting with cardiac tamponade.

**Table 1:** Trends and clinical characteristics of patients with right ventricular lead-associated perforation, including the present case M: male, F: female, ICD: implantable cardioverter defibrillator, RV: right ventricular, TLE: transvenous pacemaker lead extraction, PM: pacemaker, RVOT: right ventricular outflow tract, CRT-D: cardiac resynchronization therapy defibrillator

Subject	Age (years)	Sex	Device	Perforating lead	Perforated chamber	Lead type	Time since implantation (years)	Presenting complaint	Pretreatment condition	Pericardial effusion	Treatment	Author
1	40	M	ICD	RV	RV	Active fixation	1.3	Intermittent left shoulder pain	Unknown	+	TLE	Sharma et al. [5]
2	53	F	PM	RV	RV	Active fixation	1.7	Chest pain	Unknown	Unknown	TLE	Rajkumar et al. [6]
3	67	F	PM	RV	RV	Active fixation	2	Chest pain, palpitations	Stable	-	Open surgery (lead extraction), transvenous lead reimplantation	Yamamoto et al. [7]
4	64	M	PM	RV	RVOT	Active fixation	2.1	Chest pain	Unknown	Unknown	TLE	Rajkumar et al. [6]
5	67	M	CRT-D	RV	RVOT	Active fixation	4	Dyspnea, chest pain, tamponade	Unknown	+	Pericardiocentesis and TLE	Rajkumar et al. [6]
6	84	F	PM	RV	RV	Active fixation	4.8	Syncope	Stable	+	Open surgery (lead extraction and myocardium repair), transvenous lead reimplantation	Alla et al. [8]
7	80	F	ICD	RV	RV	Active fixation	6	Diaphoresis, dizziness	Stable	+	Pericardiocentesis and TLE, reimplantation of the ICD	Jain et al. [9]
8	79	F	PM	RV	RV	Passive fixation	9	Dyspnea	Stable	-	Open surgery (lead extraction and reimplantation of epicardial lead)	Hamada et al. [10]
Present case	60	M	PM	RV	RV	Active fixation	8	Dyspnea, chest pain, tamponade	Hemodynamically unstable	+	Open surgery without lead extraction	Nakamura et al.

The development of delayed pacemaker lead-associated perforation long after implantation due to cardiac tamponade, as in our patient, is rare. Because the right ventricle and atrium are low-pressure systems, bleeding to the extent of cardiac tamponade is unlikely to develop even if lead-associated perforation occurs. However, in our patient, the coronary vein on the right ventricular surface was accidentally perforated by the tip of the pacemaker lead, which may have caused the cardiac tamponade. This phenomenon is presumed to be possibly due, at least in part, to continuous pressure on the right ventricle from excessive bending of the ventricular lead. Compared with the eight previously reported cases (Table 1), our patient had risk factors for pacemaker lead perforation, such as the use of a screw-in lead and excessive bending of the lead, but did not present with complete perforation as described in other reports. In contrast, in our case, the lead tip had accidentally penetrated a superficial cardiac vein, which was considered to be the source of bleeding. This suggests that cardiac tamponade can occur even in the absence of complete myocardial perforation.

Currently, a clear treatment strategy for pacemaker-associated perforation has not been defined, and the decision to use transvenous treatment or open surgery should be made cautiously. Several reports indicate that pericardiocentesis and transvenous lead extraction can be used to treat right ventricular lead-associated perforation during the acute phase or at most several months from the implantation, even if the lead has penetrated the myocardium or cardiac tamponade has developed [15-17]. However, treatment strategies for delayed right ventricular lead-associated perforation occurring years after the implantation have not been adequately investigated. Some reports recommend surgical treatment with excellent direct visualization for the risk of myocardial fistula formation and bleeding [18,19]. In our patient, precise localization of the lead tip was limited by halation artifacts on CT examination, and penetration of the right ventricular wall could not be confirmed. Both CT and transthoracic echocardiography have inherent limitations in diagnostic accuracy when vascular injury on the cardiac surface is the underlying cause, as in the present case. Consequently, determining the exact cause of the cardiac tamponade was difficult. Emergency pericardial drainage was required because the patient presented with cardiac tamponade accompanied by shock vital signs, which further restricted the ability to establish the cause preoperatively. Therefore, to manage the unexpected situation and confirm the source of bleeding, we performed open surgery via a median sternotomy to decompress the tamponade.

In the previously reported cases (Table 1), including our patient, five of the nine cases underwent pericardiocentesis with transvenous pacemaker lead extraction (TLE) or TLE alone, while four cases underwent open surgery. Among the five cases in which pericardial effusion was clearly demonstrated, one underwent TLE alone, two underwent pericardiocentesis with TLE, and two underwent open surgery. Although the preoperative conditions varied, all patients recovered. Even in cases of long-delayed pacemaker lead-associated perforation, transvenous therapy appears to be a feasible treatment option. On the other hand, open surgery has the significant advantage of allowing direct visualization of the lesion.

In the management of pacemaker leads, when a lead completely perforates the right ventricular wall, removal is essential, and in many cases, reinsertion is also required. However, in our patient, the innominate vein through which the pacemaker lead had passed was strongly adherent, and removal of the lead was deemed difficult due to the risk of vascular injury. Because the pacemaker lead had not completely penetrated the right ventricular wall and its threshold and impedance had not changed, we decided to retain the same lead. However, during the postoperative period, the lead threshold increased. The reasons for this elevation were considered as follows: (i) edema of the peri-lead tissue due to inflammation around the perforation site, (ii) partial myocardial tissue necrosis around the lead caused by the purse-string suture, and (iii) further lead migration. In our patient, the elevation of the lead threshold was mild, and it could be managed by adjusting the pacemaker setting. However, in this case, although the immediate postoperative lead parameters were unchanged from the preoperative values and the device was functioning normally, subsequent elevation of the lead threshold resulted in pacing failure. Therefore, we should have implanted a temporary pacemaker lead in anticipation of a stronger change in the lead’s ambient environment. In such emergency cases, even if lead removal is not possible or deemed unnecessary, implantation of a temporary pacemaker lead in anticipation of postoperative lead failure may be necessary.

## Conclusions

Treatment strategies for long-delayed right ventricular lead-associated perforation are still being developed. Herein, we report a rare case of delayed lead-associated perforation, with vital signs indicating shock, which was treated surgically. Our experience indicates that we need to consider the unique myocardial characteristics of late-stage lead-associated perforation (e.g., myocardial fibrosis induced by long-term mechanical irritation from the lead with fistula formation at the perforation site, adhesion to surrounding tissues, and tissue fragility) when planning treatment. Furthermore, in cases where lead extraction is not performed for any reason (particularly in patients who are highly pacemaker-dependent), the placement of a temporary pacing lead should be actively considered. In other reports, the treatment plan was determined by examining each patient’s preoperative conditions. As in our case, however, there may be limitations in the pathological findings that can be identified by preoperative assessment. Further evaluation of the sequence of criteria from preoperative evaluation to treatment decisions, as well as of therapeutic options for long-term delayed pacemaker lead perforation (e.g., open surgery versus pericardiocentesis and transvenous lead extraction), is necessary to establish a general treatment plan.

## References

[1] Mond HG, Proclemer A (2011). The 11th world survey of cardiac pacing and implantable cardioverter-defibrillators: calendar year 2009--a World Society of Arrhythmia's project. Pacing Clin Electrophysiol.

[2] Herr MJ, Cottrell JM, Garrett HE Jr, Weiman DS (2020). Erosion of a right ventricular pacer lead into the left chest wall. Surg Case Rep.

[3] Lin YS, Chen TH, Hung SP (2015). Impact of pacemaker lead characteristics on pacemaker related infection and heart perforation: a nationwide population-based cohort study. PLoS One.

[4] Waddingham PH, Elliott J, Bates A (2022). Iatrogenic cardiac perforation due to pacemaker and defibrillator leads: a contemporary multicentre experience. Europace.

[5] Sharma SK, Weinsaft JW, Ip JE, Cheung JW (2016). Delayed cardiac perforation of the Durata implantable cardioverter-defibrillator lead more than 1 year after implantation. HeartRhythm Case Rep.

[6] Rajkumar CA, Claridge S, Jackson T (2017). Diagnosis and management of iatrogenic cardiac perforation caused by pacemaker and defibrillator leads. Europace.

[7] Yamamoto A, Takahashi S (2022). Delayed right ventricular lead perforation by a pacemaker lead 2-year post-implantation. Clin Case Rep.

[8] Alla VM, Reddy YM, Abide W, Hee T, Hunter C (2010). Delayed lead perforation: can we ever let the guard down?. Cardiol Res Pract.

[9] Jain S, Clancy J, Schoenfeld MH (2022). An unusual presentation of delayed lead perforation: it's never too late. HeartRhythm Case Rep.

[10] Hamada Y, Sakaki M, Watanabe Y, Hata S, Kimura K, Sakagoshi N (2023). Delayed right ventricular pacemaker lead perforation 9 years after implantation. Am J Case Rep.

[11] Issa ZF, Issa TZ (2021). Feasibility and safety of percutaneous lead revision for subacute and delayed cardiac device lead perforation. JACC Clin Electrophysiol.

[12] Hirschl DA, Jain VR, Spindola-Franco H, Gross JN, Haramati LB (2007). Prevalence and characterization of asymptomatic pacemaker and ICD lead perforation on CT. Pacing Clin Electrophysiol.

[13] Singhal S, Cooper JM, Cheung AT, Acker MA (2007). Images in cardiovascular medicine. Rib perforation from a right ventricular pacemaker lead. Circulation.

[14] Katoh M, Sugimura Y (2014). Pacemaker lead perforation through the right ventricle. Intern Med.

[15] Cano Ó, Andrés A, Alonso P, Osca J, Sancho-Tello MJ, Olagüe J, Martínez-Dolz L (2017). Incidence and predictors of clinically relevant cardiac perforation associated with systematic implantation of active-fixation pacing and defibrillation leads: a single-centre experience with over 3800 implanted leads. Europace.

[16] Laborderie J, Barandon L, Ploux S (2008). Management of subacute and delayed right ventricular perforation with a pacing or an implantable cardioverter-defibrillator lead. Am J Cardiol.

[17] Mori H, Kato R, Ikeda Y (2020). Percutaneous simple lead traction is a feasible and effective method for right ventricular lead perforations. Int Heart J.

[18] Noguchi M, Nakai T, Kawano Y, Shibayama K, Obunai K, Tabata M, Watanabe H (2017). Delayed right ventricular defibrillation lead perforation presenting as cardiac tamponade and treated surgically. Clin Case Rep.

[19] Krivan L, Kozák M, Vlasínová J, Sepsi M (2008). Right ventricular perforation with an ICD defibrillation lead managed by surgical revision and epicardial leads--case reports. Pacing Clin Electrophysiol.

